# Sex-specific modulation of juvenile social play behavior by vasopressin and oxytocin depends on social context

**DOI:** 10.3389/fnbeh.2014.00216

**Published:** 2014-06-16

**Authors:** Remco Bredewold, Caroline J. W. Smith, Kelly M. Dumais, Alexa H. Veenema

**Affiliations:** Neurobiology of Social Behavior Laboratory, Department of Psychology, Boston CollegeChestnut Hill, MA, USA

**Keywords:** female, juvenile, lateral septum, male, oxytocin receptor, play-fighting, sex difference, V1a receptor

## Abstract

We recently demonstrated that vasopressin (AVP) in the lateral septum modulates social play behavior differently in male and female juvenile rats. However, the extent to which different social contexts (i.e., exposure to an unfamiliar play partner in different environments) affect the regulation of social play remains largely unknown. Given that AVP and the closely related neuropeptide oxytocin (OXT) modulate social behavior as well as anxiety-like behavior, we hypothesized that these neuropeptides may regulate social play behavior differently in novel (novel cage) as opposed to familiar (home cage) social environments. Administration of the specific AVP V1a receptor (V1aR) antagonist (CH_2_)_5_Tyr(Me^2^)AVP into the lateral septum enhanced home cage social play behavior in males but reduced it in females, confirming our previous findings. These effects were context-specific because V1aR blockade did not alter novel cage social play behavior in either sex. Furthermore, social play in females was reduced by AVP in the novel cage and by OXT in the home cage. Additionally, females administered the specific OXT receptor antagonist desGly-NH_2_,d(CH_2_)_5−_[Tyr(Me)^2^,Thr^4^]OVT showed less social play in the novel as compared to the home cage. AVP enhanced anxiety-related behavior in males (tested on the elevated plus-maze), but failed to do so in females, suggesting that exogenous AVP alters social play and anxiety-related behavior via distinct and sex-specific mechanisms. Moreover, none of the other drug treatments that altered social play had an effect on anxiety, suggesting that these drug-induced behavioral alterations are relatively specific to social behavior. Overall, we showed that AVP and OXT systems in the lateral septum modulate social play in juvenile rats in neuropeptide-, sex- and social context-specific ways. These findings underscore the importance of considering not only sex, but also social context, in how AVP and OXT modulate social behavior.

## Introduction

Social play (also referred to as play-fighting or rough-and-tumble play) is predominantly displayed among juveniles of both sexes across many mammalian species (Bekoff and Byers, 1998; Pellis and Iwaniuk, 2000; Burghardt, 2005). These social play activities seem to contribute to the development of social and emotional skills needed throughout life, as shown in humans, non-human primates, and rodents (Sigman and Ruskin, 1999; Guralnick et al., 2006; Cordoni and Palagi, 2011). Social play deficits are observed in neurodevelopmental disorders such as autism spectrum disorders (ASD), early-onset schizophrenia, and attention-deficit/hyperactivity disorder (Alessandri, 1992; Moller and Husby, 2000; Jordan, 2003). Likewise, social play activities were found to be altered in juvenile male rats that were exposed to either maternal separation (Veenema and Neumann, 2009) or prenatal immune stress (Taylor et al., 2012). Therefore, identifying the neurochemical substrates involved in the regulation of juvenile social play behavior will increase our understanding of normal as well as impaired social development.

Among other neurochemical substrates, arginine vasopressin (AVP) and oxytocin (OXT) are two likely candidates to regulate juvenile social play. AVP and OXT have robust neuromodulatory effects on adult social behavior, including aggression, social recognition, and pair-bonding (Donaldson and Young, 2008; Veenema and Neumann, 2008; Goodson and Thompson, 2010), but less is known about their role in juvenile social behaviors, such as social play. Furthermore, AVP and OXT systems in the brain are sexually dimorphic (De Vries and Panzica, 2006; Dumais et al., 2013a). For example, males have more AVP-expressing cells in the bed nucleus of the stria terminalis and medial amygdala and denser AVP-axonal projections to limbic brain regions, especially to the lateral septum (De Vries et al., 1981; Van Leeuwen et al., 1985; Szot and Dorsa, 1993). This sexually dimorphic AVP system is already present in juveniles (De Vries et al., 1981) and is found in many mammalian species (De Vries and Panzica, 2006). Furthermore, adult male rats have higher OXT receptor (OTR) binding densities in various forebrain regions, including the lateral septum (Dumais et al., 2013a). These findings suggest that AVP and OXT, acting on, e.g., the lateral septum, may modulate social behavior, and possibly juvenile social play, in sexually dimorphic ways.

Indeed, we recently showed that pharmacological blockade of AVP V1a receptors in the lateral septum increased social play in juvenile males while it reduced social play in juvenile females (Veenema et al., 2013). In line with this, a negative correlation was found between AVP mRNA expression in the bed nucleus of the stria terminalis and social play in juvenile males (Paul et al., 2014). It is unknown, however, whether OXT via OTR in the lateral septum modulates social play behavior, and if so, whether it acts in sex-specific ways. Therefore, our first aim was to determine whether acute pharmacological manipulations of the OXT system in the lateral septum alter social play behaviors in juvenile male and female rats. Given its role in facilitating pro-social behaviors, such as mother-infant bonding, filial attachment, and pair-bonding (Williams et al., 1992; Feldman et al., 2007; Kojima and Alberts, 2011; Feldman, 2012), we predicted that OXT would enhance social play behavior and that it may do so in sex-specific ways.

In addition, little is known about the impact of contextual factors on how AVP and OXT influence social behaviors. Indeed, social play behavior in rodents typically has been tested in only one social context, i.e., exposure to a play partner in either a home cage or a novel cage setting. Understanding how different social contexts modify the social effects of AVP and OXT may inform appropriate use of AVP- and OXT-based drugs to restore social function (Bartz et al., 2011) since both neuropeptides have been implicated in ASD and other social disorders (Meyer-Lindenberg et al., 2011; Modi and Young, 2012) and are currently being tested in clinical trials (clinicaltrials.gov). Therefore, our second aim was to determine whether AVP and OXT regulate social play differently in novel as opposed to familiar environments. We hypothesized that exposure to an unfamiliar rat in the novel environment (novel cage) vs. a familiar environment (home cage) would alter the salience of the stimulus rat, and thus, result in a differential regulation of social play by AVP and OXT. Because AVP typically enhances (Landgraf et al., 1995; Beiderbeck et al., 2007) while OXT reduces (Windle et al., 1997; Ring et al., 2006; Blume et al., 2008) anxiety-like behaviors of rats exposed to novel environments, we predicted that AVP would reduce social play while OXT would increase social play in the novel cage. To determine whether drug-induced alterations in social play were associated with alterations in anxiety-like behavior, we exposed rats to the elevated plus-maze to measure non-social anxiety-like behavior.

To study social context-specific roles of AVP and OXT in the modulation of juvenile social play behavior, the lateral septum was chosen as the region of interest because (1) this area serves as a hub in the brain by integrating social, emotional, and contextual information from different brain systems and contributing to the coordination of adaptive context-specific behavioral responses (Sheehan et al., 2004; Luo et al., 2011), (2) V1aR and the OTR are abundantly expressed in this area in juvenile rats (Lukas et al., 2010; Veenema et al., 2012), and (3) AVP via V1aR in the lateral septum modulates social play behavior in the home cage (Veenema et al., 2013).

## Materials and methods

### Animals

Male and female Wistar rats (23-day-old) were obtained from Charles River (Raleigh, NC) and maintained under standard laboratory conditions (12 h light/dark cycle, lights off at 14:00 h, 22°C, 50% humidity, food and water *ad libitum*). Rats were housed in same-sex groups of four in standard rat cages (48 × 27 × 20 cm) unless otherwise mentioned. The experiments were conducted in accordance with the guidelines of the NIH and approved by Boston College Institutional Animal Care and Use Committee.

### Cannulation

**Figure 1:**
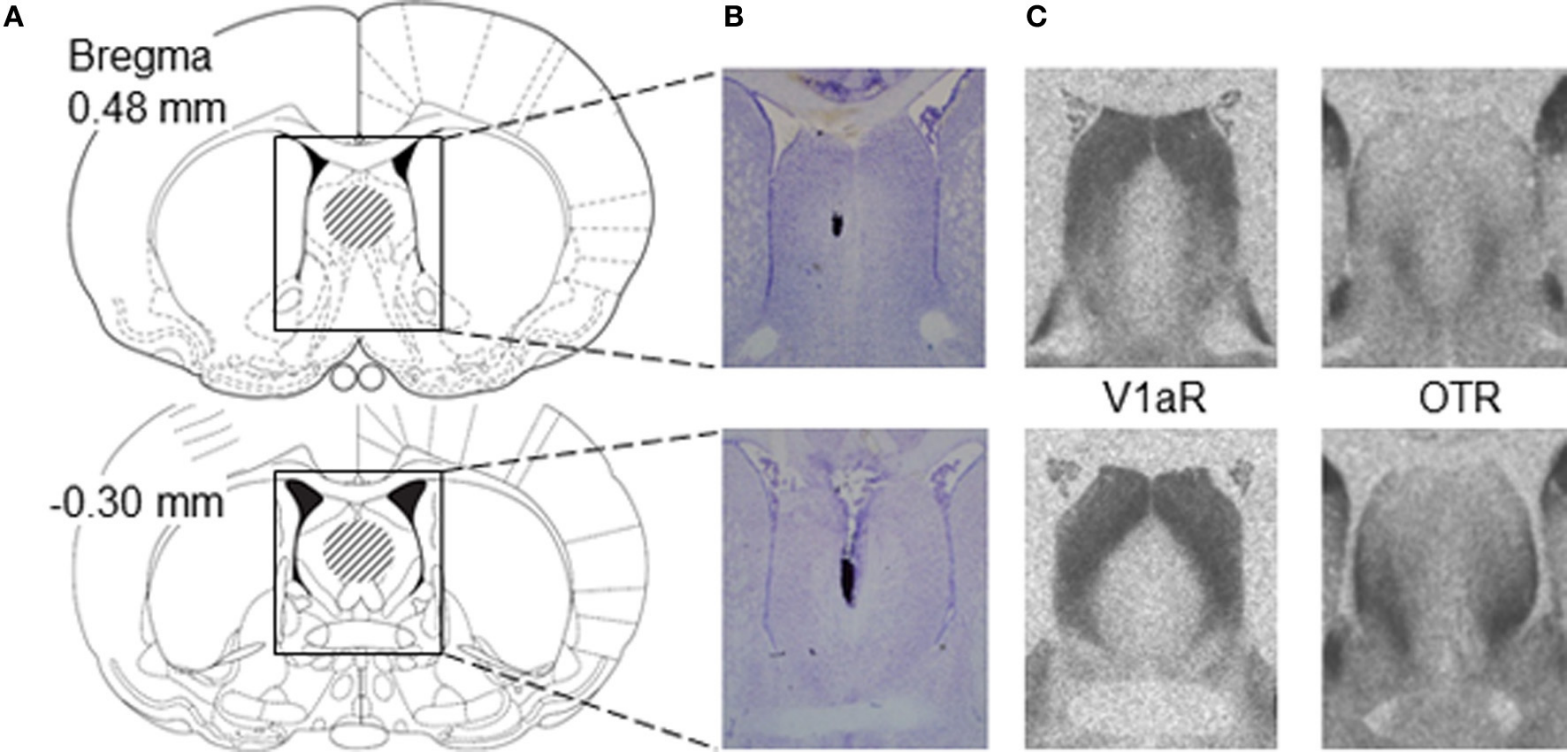
**Placement of microinjections relative to V1aR and OTR binding in the lateral septum of juvenile rats. (A)** Schematic diagrams of coronal rat brain sections depicting the lateral septum (Paxinos and Watson, 1998). The dashed areas in the diagrams indicate the area of correct placement of microinjections. Microinjection placement in the lateral septum was considered correct between bregma distances 0.48 and −0.30 mm according to Paxinos and Watson (1998). **(B)** Representative photomicrographs of Nissl-stained coronal brain sections showing correct microinjection placement into the medial part of the lateral septum based on charcoal deposits. **(C)** Photomicrographs represent autoradiograms of 16 μm coronal brain sections showing V1aR binding and OTR binding in the lateral septum of a juvenile male rat.

After 1 week of daily handling to familiarize rats with the injection procedure, juvenile (30-31 days of age) males and females were anesthetized with isoflurane (Butler Schein Animal Health, Dublin, OH) and mounted on a stereotaxic frame with the tooth bar set at −4.5 mm. Single guide cannula (22 gauge; Plastics One, Roanoke, VA) were implanted stereotaxically 2 mm dorsal to the medial part of the lateral septum at coordinates 0.4 mm caudal to bregma (corresponding with Figures 16–19 of the adult rat brain atlas of Paxinos and Watson, 1998; see also Figure 1), −1.0 mm lateral to the midline and 3.6 mm ventral to the surface of the skull under an angle of 10° from the midsagittal plane to avoid damage to the sagittal sinus. This injection site was chosen because it is positioned centrally in the region where males and females express high densities of V1aR and OTR binding in the lateral septum (Lukas et al., 2010; Veenema et al., 2012; Figure 1) and because the injection site corresponds with previous studies showing that pharmacological manipulations of AVP and OXT systems in the rat lateral septum altered social behavior (Veenema et al., 2010, 2012, 2013; Lukas et al., 2013), indicating that the drugs are reaching their target receptors. Cannulae were fixed to the skull with two stainless steel screws and dental cement and closed with a dummy cannula (Plastics One, Roanoke, VA).

### Familiar and novel environments

After surgery, rats were individually housed in standard rat cages (48 × 27 × 20 cm). Bedding in the home cage was not changed over the course of the experiment (i.e., 5 days). The first set of rats was tested for social play 3 and 5 days after surgery, once in the home cage and once in a novel cage in counterbalanced order, and given the same drug. The home and novel cage were chosen as familiar and novel environments because rats are exposed to both environments when being reared in animal facilities making them realistic environments. The second set of rats was tested for non-social anxiety on the elevated plus-maze, 3 days after surgery. The 3-day recovery period matches that used for other procedures applied routinely in our lab (e.g., intracerebral microdialysis) and was shown not to affect the display of social behavior (Beiderbeck et al., 2007; Veenema et al., 2010, 2012; Lukas et al., 2011, 2013).

### Social play test

During the beginning of the dark phase (between 14:00 and 15:00 h) and under red light conditions, experimental rats were exposed to an age- and sex-matched unfamiliar rat for a period of 10 min. All tests were videotaped for subsequent analysis of behavior by a researcher blind to the treatment condition using JWatcher (http://www.jwatcher.ucla.edu/). The following behaviors were scored for the experimental rat according to Veenema et al. (2013): nape attack (the experimental rat displays nose attacks or nose contacts toward the nape of the neck of the stimulus rat), pinning (the experimental rat holds the stimulus rat on its back), supine (the experimental rat lays on its back and is pinned by the stimulus rat), social play (the total amount of time spent in playful social interactions including nape attacks, pinning, and supine poses), social investigation (the experimental rat is sniffing the stimulus rat), non-social exploration, and allo-grooming (the experimental rat is grooming the stimulus rat).

### Elevated plus-maze test

The elevated plus-maze is based on the rat's natural conflict between its exploratory drive and its innate fear of elevated, open and novel spaces (Pellow et al., 1985). It consists of two opposing open (50 × 10 cm; 60 lux) and two opposing closed (50 × 10 × 40 cm; 20 lux) arms, which are connected by a common central area (10 × 10 cm). A raised edge (0.5 cm) on the open arms provided additional grip for the rats. The apparatus is made of dark gray plastics and was elevated to a height of 90 cm above the floor. Tests took place in the late light phase (between 10:00 and 12:00 h). Before each trial, the maze was cleaned with water containing a low concentration of detergent. Rats were placed individually in the center square facing a closed arm and were allowed to explore the maze for 5 min. Behavior was measured by means of a video camera mounted above the maze and scored in an adjacent room by a researcher blind to the treatment condition using JWatcher. An open/closed arm entry was defined as both forepaws and shoulders of the rat being on the respective arm of the elevated plus-maze. The percentage of time spent on the open arms [100 × time on open arms/(time on open arms + time in closed arms)] and the percentage of open arm entries [100 × open arm entries/(open + closed arm entries)] were used as parameters of anxiety-like behavior. The number of total (closed and open arm) entries was used as a measure of locomotor activity.

### Microinjections

**Figure 2:**
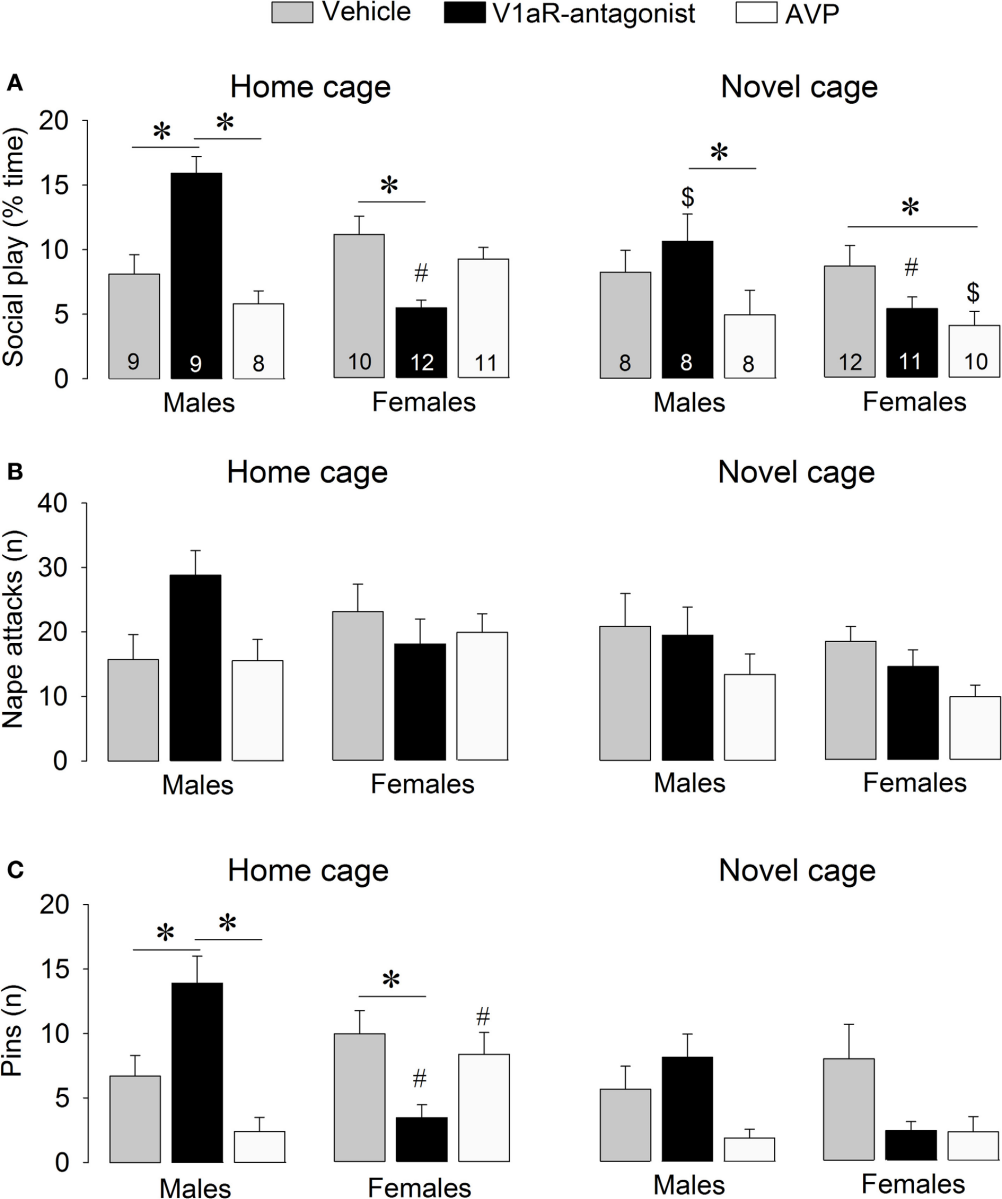
**Effects of acute pharmacological manipulations of the AVP system in the lateral septum on the duration of social play **(A)**, the number of nape attacks **(B)**, and the number of pins **(C)** in male and female juvenile rats exposed to an age- and sex-matched unfamiliar rat in either the home cage or a novel cage**. Bars indicate mean + s.e.m. ^*^p < 0.05 (treatment effect), ^#^*p* < 0.05 vs. male counterparts (sex effect), ^$^*p* < 0.05 vs. home cage (context effect). The *p*-values are indicated as <0.05 for simplicity (see text for details); Three-Way ANOVA followed by Bonferroni *post-hoc* tests (Social play) or by Two-Way ANOVA and Bonferroni *post-hoc* tests (Pins).

**Figure 3:**
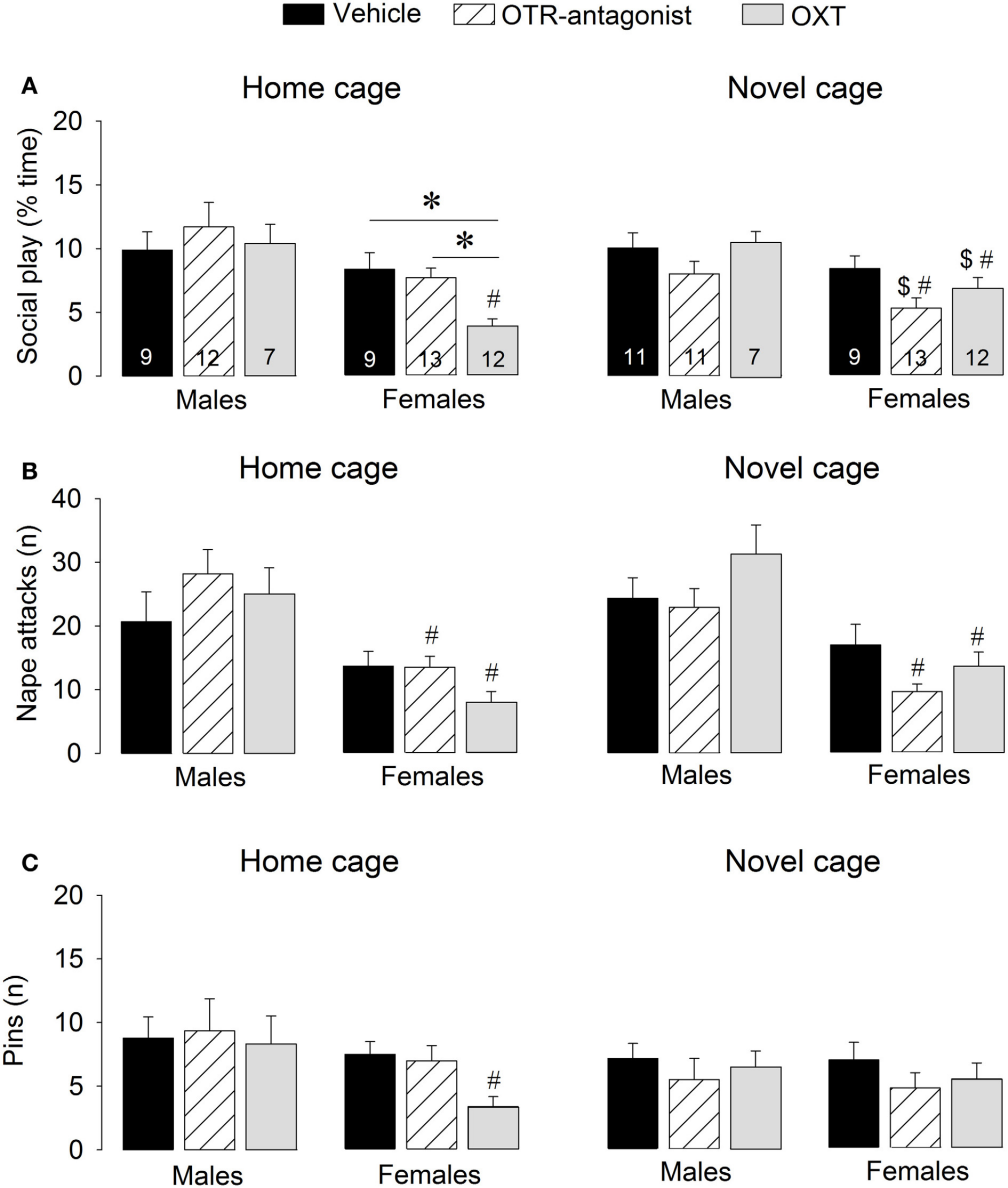
**Effects of acute pharmacological manipulations of the OXT system in the lateral septum on the duration of social play **(A)**, the number of nape attacks **(B)**, and the number of pins **(C)** in male and female juvenile rats exposed to an age- and sex-matched unfamiliar rat in either the home cage or a novel cage**. Bars indicate mean + s.e.m. ^*^p < 0.05 (treatment effect), ^#^*p* < 0.05 vs. male counterparts (sex effect), ^$^*p* < 0.05 vs. home cage (context effect). The *p*-values are indicated as <0.05 for simplicity (see text for details); Three-Way ANOVA followed by Two-Way ANOVA's and Bonferroni *post-hoc* tests.

**Figure 4:**
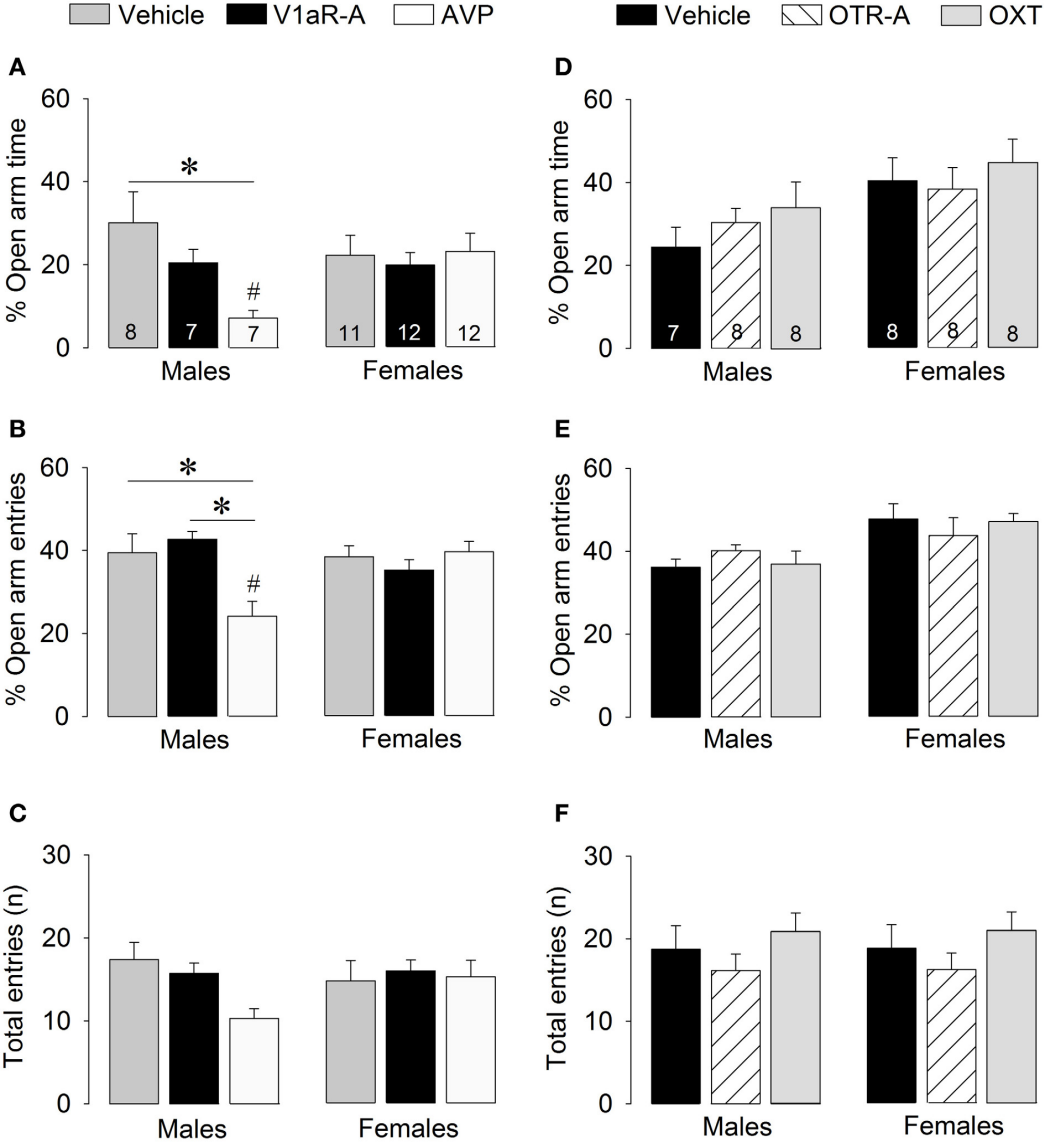
**Effects of acute pharmacological manipulations of the AVP or OXT system in the lateral septum on anxiety-related behaviors (A,B,D,E) and locomotion (C,F) in male and female juvenile rats tested on the elevated plus-maze**. Bars indicate mean + s.e.m. ^*^p < 0.05 (treatment effect), ^#^*p* < 0.05 vs. female counterparts (sex effect). The *p*-values are indicated as < 0.05 for simplicity (but see text for details); Two-Way ANOVA followed by Bonferroni *post-hoc* tests.

Selective V1aR and OTR antagonists were used to determine the role of the AVP and OXT receptor systems, respectively, in social play behavior. The effects of exogenous AVP and OXT on social play behavior were also determined. In detail, rats received an injection 20 min prior to either the social play test or the elevated plus-maze test. The injection was given over a period of 1 min into the middle part of the lateral septum via an injector cannula that extended 2 mm beyond the guide cannula and was connected via polyethylene tubing to a Hamilton syringe. The injector cannula was kept in place for 30 s following injection to allow for tissue uptake. The injector cannula was then replaced by a dummy cannula. Rats received either 0.5 μl of Ringer's solution (vehicle), the specific V1aR antagonist d(CH_2_)_5_[Tyr(Me)^2^]AVP (10 ng/0.5 μl; V1aR-A; Manning et al., 2008), synthetic AVP (200 pg/0.5 μl; Sigma-Aldrich), the specific OTR antagonist desGly-NH_2_,d(CH_2_)_5−_[Tyr(Me)^2^,Thr^4^]OVT (10 ng/0.5 μl; OTR-A; Manning et al., 2008), or synthetic OXT (200 pg/0.5 μl; Sigma-Aldrich). These drug concentrations were shown to be effective in altering social play (Veenema et al., 2013) or other social behaviors (Veenema et al., 2012; Dumais et al., 2013b; Lukas et al., 2013) in rats. At the end of the experiments, rats were killed with CO_2_, and charcoal was injected as a marker to check proper placement of the cannulae into the lateral septum histologically on Nissl-stained coronal brain sections. The final number of rats for each group is indicated in the first row of graphs in Figures 2–4.

### Statistics

Social play behaviors were analyzed using a Three-Way ANOVA (sex × treatment × context). Bonferroni *post-hoc* tests, Two-Way ANOVAs, or One-Way ANOVAs were used to test for differences among groups. Elevated plus-maze behaviors were analyzed using a Two-Way ANOVA (sex × treatment), followed by Bonferroni *post-hoc* tests to test for differences among groups. Data are presented as mean ± s.e.m. Significance was set at *p* < 0.05. In all figures, a significant treatment effect is indicated by the symbol ^*^, a significant sex effect is indicated by the symbol #, and a significant context effect is indicated by the symbol $.

## Results

### Modulation of social play behavior by AVP

V1aR-A treatment enhanced social play behaviors in males, but reduced these behaviors in females. These effects were context-specific as they appeared in the home cage but not in the novel cage. AVP treatment reduced social play in females, but only in the novel cage. Details on main effects and details on *post-hoc* and One-Way ANOVA tests are given in Table 1.

**Table 1:** **Significant main effects on behavior in the social play test in response to AVP system manipulations in the lateral septum**.

Treatment × Sex × Context	Social play	*F*_(2, 104)_ = 3.96, *p* < 0.05
	Allo-grooming	*F*_(2, 104)_ = 3.46, *p* < 0.05
	Exploration	*F*_(2, 104)_ = 6.86, *p* < 0.005
Treatment × Sex	Pins	*F*_(2, 104)_ = 14.44, *p* < 0.001^*^
Sex	Supine	*F*_(2, 104)_ = 8.96, *p* < 0.005
Context	Nape attacks	*F*_(1, 104)_ = 4.18, *p* < 0.05

* *Subsequent Two-Way ANOVA's were run to examine the effects of treatment and sex independently for the two contexts. This yielded a significant treatment × sex effect in the home cage only [F_(2, 53)_ = 15.3, p < 0.001]*.

#### Treatment effects

V1aR-A treatment in the home cage enhanced social play in males (*p* < 0.001 vs. vehicle-treated and vs. AVP-treated males, Figure 2A) while it reduced social play in females (*p* < 0.01 vs. vehicle-treated females, Figure 2A). V1aR-A treatment in the home cage enhanced the number of pins in males (*p* < 0.05 vs. vehicle-treated and *p* < 0.001 vs. AVP-treated males, Figure 2C) while it reduced the number of pins in females (*p* < 0.05 vs. vehicle-treated females, Figure 2C). AVP treatment in the novel cage reduced social play in females (*p* < 0.05 vs. vehicle-treated females, Figure 2A) while in males this was only significant vs. V1aR-A-treated males (*p* < 0.05, Figure 2A). Treatment effects were also seen for non-social exploration (Table S1), which were likely secondary to the V1aR-A and AVP treatment effects on social play.

#### Sex effects

Vehicle-treated male and female rats did not show sex differences in social play behaviors (Figure 2, Table S1). V1aR-A-treated females showed less social play (*p* < 0.001 home cage, *p* < 0.01 novel cage, Figure 2A) and fewer pins (*p* < 0.001 home cage, Figure 2C) than V1aR-A-treated males. In contrast, AVP-treated females in the home cage showed more pins (*p* < 0.05, Figure 2C) than AVP-treated males. Additional sex effects were found for supine (Table 1, allo-grooming (Table S1), and non-social exploration (Table S1).

#### Context effects

Social play behaviors in vehicle-treated rats did not differ between home and novel cage. However, V1aR-A-treated males showed less social play (*p* < 0.01, Figure 2A) in novel vs. home cage. Moreover, AVP-treated females showed less social play (*p* < 0.005, Figure 2A) in novel vs. home cage. Additional context effects were found for nape attacks (Table 1), allo-grooming (Table S1), and non-social exploration (Table S1).

### Modulation of social play behavior by OXT

OTR-A treatment did not alter social play behaviors. However, OXT reduced social play, but only in females and only in the home cage. Details on main effects and details on *post-hoc* and One-Way ANOVA tests are given in Table 2.

**Table 2:** **Significant main effects on behavior in the social play test in response to OXT system manipulations in the lateral septum**.

Treatment × Context	Social play	*F*_(2, 113)_ = 4.12, *p* < 0.05^*^
	Nape attacks	*F*_(2, 113)_ = 3.64, *p* < 0.05^*^
Sex	Social play	*F*_(1, 113)_ = 25.2, *p* < 0.001^#^
	Nape attacks	*F*_(1, 113)_ = 55.3, *p* < 0.001^#^
	Pins	*F*_(1, 113)_ = 3.93, *p* = 0.050^#^

* Subsequent Two-Way ANOVA's were run to examine the effects of treatment and context separately by sex. This yielded a significant treatment × context effect in females only [social play: F_(2, 62)_ = 5.01, p < 0.05; nape attacks: F_(2, 62)_ = 3.15, p = 0.050].

# *Subsequent One-Way ANOVA's were run to examine the sex effect separately for treatment and context. This yielded several sex effects (see text for details)*.

#### Treatment effects

OTR-A-treated rats did not differ from vehicle-treated rats in any behavior in the social play test (Figure 3, Table S2). In contrast, OXT-treated females in the home cage showed less social play than vehicle-treated females (*p* < 0.005) and OTR antagonist-treated females (*p* < 0.01, Figure 3A). No treatment effects were found for males or for other behaviors (Table S2).

#### Sex effects

Vehicle-treated rats did not show a sex difference in social play behaviors in home or novel cage (Figure 3, Table S2). OTR-A-treated females displayed less social play (*p* < 0.05 novel cage, Figure 3A) and fewer nape attacks (*p* < 0.001 home cage, *p* < 0.001 novel cage, Figure 3B) than OTR-A-treated males. OXT-treated females displayed less social play (*p* < 0.001 home cage, *p* < 0.05 novel cage, Figure 3A), fewer nape attacks (*p* < 0.001 home cage, *p* < 0.005 novel cage, Figure 3B) and fewer pins (*p* < 0.05, home cage, Figure 3C) than OXT-treated males. No sex effects were found for other behaviors (Table S2).

#### Context effects

Social play behaviors in vehicle-treated rats did not differ between home and novel cage. However, OTR-A-treated females showed less social play (*p* < 0.05) in novel vs. home cage (Figure 3A) while OXT-treated females showed more social play (*p* < 0.05, Figure 3A) in novel vs. home cage. No context effects were found for males or for other behaviors (Table S2).

### Modulation of non-social anxiety-like behavior by AVP

V1aR-A treatment did not alter anxiety-like behaviors on the elevated plus-maze. However, AVP treatment altered anxiety in sex-specific ways as reflected by treatment x sex effects for percentage time on open arms [*F*_(2, 51)_ = 3.41, *p* < 0.05] and percentage open arm entries [*F*_(2, 51)_ = 7.39, *p* < 0.05]. *Post-hoc* testing indicates that AVP-treated males spent less time on the open arms (Figure 4A) and had less open arm entries (Figure 4B) than vehicle-treated males (time: *p* < 0.01; entries: *p* < 0.01) and V1aR-A-treated males (entries: *p* < 0.005). Furthermore, AVP-treated males spent less time on the open arms (*p* < 0.05, Figure 4A) and had less open arm entries (*p* < 0.005, Figure 4B) than AVP-treated females. No main effects were found for locomotion (total arm entries; Figure 4C).

### Modulation of non-social anxiety-like behavior by OXT

No main effects were found for any of the parameters of anxiety-like behaviors or locomotion, indicating that OTR-A and OXT treatments did not alter non-social anxiety-like behavior or locomotion on the elevated plus-maze (Figures 4D–F).

## Discussion

We have shown that AVP and OXT modulate social play behavior in juvenile rats in neuropeptide-, sex-, and social context-specific ways. Specifically, V1aR blockade enhanced social play behaviors in males, but reduced these behaviors in females. These effects were found when rats were tested in their home cage, while no such effects were found in a novel cage. Furthermore, social play behaviors in females were reduced by AVP treatment in the novel cage and by OXT treatment in the home cage. These findings indicate that sex and social context are important factors to consider in how AVP and OXT modulate social play behavior and, most likely, other social behaviors as well.

### Regulation of social play by AVP and OXT systems in the lateral septum: sex-specific effects

No sex differences were found for home cage social play behaviors in vehicle-treated rats, confirming our previous findings (Veenema et al., 2013) and those of others using a resident-intruder setting (Panksepp and Beatty, 1980; Panksepp, 1981; Thor and Holloway, 1986). In contrast, robust sex differences were found in home cage social play behaviors in response to acute pharmacological manipulations of the AVP and OXT systems in the lateral septum. Males showed more social play behaviors (duration of social play and number of pins) than females after V1aR-A treatment, confirming our previous findings (Veenema et al., 2013). Moreover, females showed fewer nape attacks than males after OTR-A treatment, supporting our previous findings when given OTR-A intracerebroventricularly (Veenema et al., 2013). Finally, females showed less social play than males after OXT treatment. Together, these findings strongly suggest that the neural mechanisms by which AVP and OXT modulate social play differ between males and females. This could be due to sex differences in either AVP/OXT release in the lateral septum, in the types of lateral septum neurons expressing the V1aR and OTR, in neuropeptide-mediated modulation of lateral septum neuronal activity, or in a combination of these. Since male rats have more AVP fibers (De Vries et al., 1981), lower V1aR binding density (Veenema et al., 2012) and higher OTR binding density (Dumais et al., 2013a) in the lateral septum than female rats, these suggestions would be worth testing. Moreover, the lateral septum projects to various brain areas that modulate motivational and behavioral responses including the lateral hypothalamus, nucleus accumbens, and ventral tegmental area (Sheehan et al., 2004). This may indicate that, by acting on the lateral septum, AVP and OXT drive sex-specific neural circuits that modulate social play. While there is a growing interest in identifying the neural circuits underlying social play behavior (Vanderschuren et al., 1997; Trezza et al., 2010; Siviy and Panksepp, 2011), our findings clearly indicate that this requires the inclusion of both sexes in future studies.

Sex-specific responses to AVP and OXT manipulations in rodents have been reported for behaviors other than juvenile social play, including aggression (Ferris and Potegal, 1988; Gutzler et al., 2010), partner preference (Insel and Hulihan, 1995; Cho et al., 1999; Bales et al., 2013), alloparental behavior (Bales et al., 2004), and social recognition (Bluthé and Dantzer, 1990; Engelmann et al., 1998; Veenema et al., 2012). Likewise, sex differences in the effects of AVP and OXT manipulations on behavioral and brain responses have been reported in adult humans (Thompson et al., 2006; Domes et al., 2007, 2010; Lischke et al., 2012; Rilling et al., 2014). These studies were performed on healthy animals and humans, but their outcomes suggest that males and females may show sex-specific susceptibility to alterations in AVP and/or OXT systems. This may be relevant for understanding sex-biases in prevalence, symptom severity, and treatment responses in neuropsychiatric disorders characterized by social dysfunction and in which AVP and OXT systems may play a role (Rutter et al., 2003; Carter, 2007). Additionally, these sex-specific behavioral responses to drugs targeting the AVP and OXT systems signify the importance of taking into consideration the sex of the individual in all studies, but especially in clinical settings when applying drugs targeting the AVP and/or OXT systems to improve social functioning (MacDonald, 2013).

### Regulation of social play by AVP and OXT systems in the lateral septum: context-specific effects

Social context by itself did not affect social play behaviors in vehicle-treated males and females. However, social context modulated the effects of AVP and OXT manipulations on social play behavior. First, the effects of V1aR blockade on social play behavior in the home cage were absent in a novel cage. Second, AVP decreased social play behavior in females in the novel but not in the home cage. Third, social play behavior in females was lower in response to OTR blockade in the novel vs. home cage. Last, OXT decreased social play behavior in females in the home but not in the novel cage.

The context-specific behavioral effects of AVP and OXT systems in the lateral septum correspond with other studies reporting that the social context modulates the behavioral responses of neuropeptide systems. For example, central blockade of the V1aR reduces aggression in dominant male violet-eared waxbills toward an unfamiliar male during mate competition aggression but not during resident-intruder aggression (Goodson et al., 2009). OXT induced flank marking when dominant female Syrian hamsters were tested with a familiar, but not with a novel, subordinate partner (Harmon et al., 2002). Moreover, blockade of the vasoactive intestinal polypeptide receptor in the brain of zebra finches alters social contact behavior in a novel, but not familiar, environment (Kingsbury et al., 2013). There are also human studies showing that the behavioral effects of AVP and OXT depend on social contextual factors (Declerck et al., 2010; Bartz et al., 2011; Rilling et al., 2012; Olff et al., 2013). These findings suggest that context-dependent actions of AVP and OXT are a common feature across different species, underscoring the importance of studying their underlying neural mechanisms.

Because of its anxiogenic effects, we predicted that AVP would reduce social play in the novel cage setting. Indeed, AVP-treated females showed less novel cage social play behavior compared to vehicle-treated females. However, AVP had no effect on anxiety-like behavior on the elevated plus-maze in juvenile females. Conversely, AVP failed to alter novel cage social play behavior in juvenile males, yet it had a strong anxiogenic effect on elevated plus-maze behavior, confirming previous findings in adult male rats (Landgraf et al., 1995; Beiderbeck et al., 2007; Veenema et al., 2010). This indicates that AVP alters novel cage social play behavior via mechanisms different from those that alter elevated plus-maze behavior.

Given the well-known role of OXT in facilitating pro-social behaviors (Williams et al., 1992; Feldman et al., 2007; Kojima and Alberts, 2011; Feldman, 2012) and reducing anxiety-like behaviors (Windle et al., 1997; Ring et al., 2006; Blume et al., 2008), we expected to find a facilitating effect of OXT on social play, especially in the novel cage. In contrast, OXT treatment did not alter social play behavior in the novel cage, but decreased female social play behaviors in the home cage. Interestingly, some rodent studies provide evidence suggesting that OXT in the lateral septum may reduce pro-social behaviors and enhance anxiety. For example, OTR binding density in the lateral septum correlated negatively with huddling behavior in female meadow voles (Beery and Zucker, 2010) and with alloparental behavior in female prairie voles (Olazábal and Young, 2006), and was lower in group-living as opposed to solitary tuco-tucos (Beery et al., 2008). This may indicate that low rather than high OTR binding in the lateral septum is important for social affiliation.

Moreover, OTR activation in the lateral septum enhanced conditioned fear responses in mice exposed to acute social defeat (Guzman et al., 2013). Likewise, exogenous OXT can have anxiogenic effects in humans exposed to social stimuli (Bartz et al., 2010; Striepens et al., 2012; Grillon et al., 2013; MacDonald et al., 2013). These findings suggests that OXT has a modulatory role in responding to the salience and/or valence of social stimuli, likely facilitating affiliative responses to positive social stimuli and anxiogenic responses to negative social stimuli. Indeed, several of the above-mentioned studies have in common that OXT potentiated fear in response to negative social stimuli. No such negative stimuli were present in our study when female juvenile rats were exposed in their home cage to an unfamiliar juvenile rat. However, OTR blockade in the lateral septum (this study) and intracerebroventricularly (Veenema et al., 2013) failed to alter social play behavior in either sex, suggesting that there is no increase in endogenous OXT release in the brain during social play behavior. Hence, exogenous OXT may have induced an artificial situation resulting in a change in the valence of the social play partner, i.e., from a positive one to a more negative one. Future studies will need to test this hypothesis and explore its underlying neural circuitry. Clearly, our results add to an emerging literature revealing the complexity of OXT effects on social behavior and signifying that OXT, at least acting on the lateral septum, can reduce pro-social behaviors, such as social play.

Social play is a highly motivational behavior (Trezza et al., 2010) and the lateral septum plays a key role in modulating context-specific motivational states (Sheehan et al., 2004; Luo et al., 2011; Sartor and Aston-Jones, 2012). Hence, the context-specific effects of V1aR antagonist, AVP, OTR antagonist, and OXT treatments on social play could have been mediated by changes in the activation of motivational systems in the brain. A possible circuit could comprise the hippocampus transmitting contextual information onto the lateral septum which, in turn, relays this information onto areas such as the lateral hypothalamus and the ventral tegmental area to modulate behavioral responses appropriate to particular environmental stimuli (Sheehan et al., 2004). In this scenario, social context-dependent input from the hippocampus to the lateral septum may have modulated the ability of AVP and OXT systems to alter lateral septum activity resulting in context-specific effects of V1aR antagonist, AVP, OTR antagonist, and OXT treatments on social play behavior. This should be tested in future studies.

Despite the context-specific effects, neither administration of AVP nor administration of OTR-A altered social play behavior in males and females tested in the home cage. We cannot exclude that higher drug doses would be effective in altering social play behavior in the home cage. However, the same dose of AVP altered novel cage social play behavior in females, anxiety-related behavior in males, and social recognition in both male and female juvenile rats (Veenema et al., 2012). Therefore, it may be that endogenous AVP already influences social play behavior at maximum levels. Likewise, the same dose of OTR-A impaired social recognition when injected into the lateral septum (Lukas et al., 2013) or in the bed nucleus of the stria terminalis (Dumais et al., 2013b), an area that shows denser OTR binding than the lateral septum (Dumais et al., 2013a). Determining the release pattern of OXT and AVP during social play behavior may further help define the involvement of these neuropeptides in the sex- and context-specific modulation of social play behavior. Additionally, the neuropeptide- and receptor antagonist-specific effects on social play suggests that it is less likely that AVP and OXT may have acted via each other's receptors (Chini and Manning, 2007; Schorscher-Petcu et al., 2010). Nevertheless, future studies administering receptor antagonists prior to the neuropeptide would be required to further confirm that the effects of AVP and OXT on social play are mediated via their “own” receptor.

In conclusion, we have shown that AVP and OXT systems in the lateral septum regulate social play behavior in juvenile rats. This regulation was found to be (1) neuropeptide-specific, indicating that AVP and OXT in the lateral septum uniquely modulate neural circuits involved in social play, (2) sex-specific, suggesting that such neural circuits differ between males and females, and (3) social context-dependent, demonstrating that generalizations cannot be made about the roles of AVP and OXT across social contexts. These findings may be informative to humans, such that AVP- and/or OXT-based treatments of social deficits may have sex- and social context-dependent effects.

### Conflict of interest statement

The authors declare that the research was conducted in the absence of any commercial or financial relationships that could be construed as a potential conflict of interest.
