# Supplementary material for: Sex-specific modulation of juvenile social play behavior by vasopressin and oxytocin depends on social context
Source: Front Behav Neurosci. 2014 Jun 16;8:216. doi: 10.3389/fnbeh.2014.00216 (PMC4058593; doi:10.3389/fnbeh.2014.00216)
Supplement: Supplementary file 1 [file DataSheet1.ZIP › Supp Table 1.pdf]

1.1. Supplementary Tables

Supplementary Table 1. Effect of manipulations of the AVP system in the lateral septum on additional behaviors in the social play test.

		Home cage		Novel cage	
		Male	Female	Male	Female
Supine	Vehicle	2.0 ± 0.8	3.5 ± 1.4	1.4 ± 0.9	3.3 ± 0.9
	V1aR-A	1.0 ± 0.3	3.4 ± 1.0	2.6 ± 1.6	3.3 ± 1.4
	AVP	2.1 ± 0.8	3.1 ± 0.7	0.8 ± 0.3	2.7 ± 1.5
Soc. Invest.	Vehicle	4.7 ± 0.7	4.9 ± 1.0	6.6 ± 0.7	4.5 ± 0.8
	V1aR-A	4.7 ± 0.8	5.8 ± 1.1	3.9 ± 0.6	5.5 ± 0.5
	AVP	3.9 ± 0.8	4.0 ± 0.6	6.3 ± 0.9	4.8 ± 0.6
Allo-groom.	Vehicle	1.4 ± 0.6	2.7 ± 1.1	0.7 ± 0.5	7.9 ± 2.2[d, h]
	V1aR-A	6.5 ± 1.6[e]	1.1 ± 0.5	3.0 ± 1.1	5.0 ± 1.9
	AVP	2.7 ± 1.6	5.0 ± 1.4	5.0 ± 2.7	3.8 ± 1.4
Exploration	Vehicle	76.5 ± 2.2	75.6 ± 1.7	77.6 ± 2.6	71.2 ± 2.3[f]
	V1aR-A	68.2 ± 1.8[a, b, g, i]	77.6 ± 1.5	77.7 ± 2.5	74.9 ± 1.9
	AVP	78.9 ± 2.5	73.1 ± 2.3	74.0 ± 2.9	78.5 ± 2.2[c]

Data represent means ± SEM of number of supine behaviors or duration (in percentage of time) of social investigation (Soc. Invest.), allo-grooming, and non-social exploration (Exploration). V1aR-A, V1aR antagonist. Treatment effects: a, $p < 0.05$ versus vehicle-treated males; b, $p < 0.005$ versus AVP-treated males; c, $p < 0.05$ versus vehicle-treated females. Sex effects: d, $p < 0.005$ versus vehicle-treated males; e, $p < 0.05$ versus V1aR-A-treated females; f, $p < 0.05$ versus vehicle-treated males; g, $p < 0.005$ versus V1aR-A-treated females. Context effects: h, $p < 0.05$ versus home cage; i, $p < 0.01$ versus novel cage. Three-way ANOVA followed by Bonferroni post-hoc tests or by two-way ANOVA and Bonferroni post-hoc tests.
