# Supplementary material for: Sex-specific modulation of juvenile social play behavior by vasopressin and oxytocin depends on social context
Source: Front Behav Neurosci. 2014 Jun 16;8:216. doi: 10.3389/fnbeh.2014.00216 (PMC4058593; doi:10.3389/fnbeh.2014.00216)
Supplement: Supplementary file 1 [file DataSheet1.ZIP › Supp Table 2.pdf]

Supplementary Table 2. No significant effects of manipulations of the OXT system in the lateral septum on additional behaviors in the social play test.

		Home cage		Novel cage	
		Male	Female	Male	Female
Supine	Vehicle	2.9 ± 0.8	1.6 ± 0.4	2.1 ± 0.7	2.0 ± 0.4
	OTR-A	1.6 ± 0.5	1.8 ± 0.5	1.2 ± 0.5	1.2 ± 0.4
	OXT	2.6 ± 1.3	1.3 ± 0.3	2.7 ± 0.6	1.9 ± 0.5
Soc. Invest.	Vehicle	4.2 ± 0.8	4.2 ± 0.7	3.5 ± 0.5	2.8 ± 0.4
	OTR-A	3.7 ± 0.8	3.7 ±0.7	3.6 ± 0.7	3.8 ± 0.4
	OXT	3.3 ± 0.9	4.3 ± 0.5	4.3 ± 1.5	4.3 ± 0.6
Allo-groom.	Vehicle	0.8 ± 0.4	4.3 ± 1.6	2.8 ± 0.8	2.8 ± 1.3
	OTR-A	3.6 ± 1.4	4.1 ± 1.5	2.8 ± 0.8	3.5 ± 1.2
	OXT	2.1 ± 1.3	4.3 ± 1.4	3.0 ± 2.6	2.4 ± 1.0
Exploration	Vehicle	78.0 ± 2.2	76.8 ± 2.8	78.7 ± 1.1	77.5 ± 1.8
	OTR-A	75.4 ± 2.8	77.9 ± 2.6	81.9 ± 1.4	81.3 ± 1.4
	OXT	80.5 ± 2.5	79.8 ± 1.9	78.1 ± 2.9	81.1 ± 1.5

Data represent means ± SEM of number of supine behaviors or duration (in percentage of time) of social investigation (Soc. Invest.), allo-grooming, and non-social exploration (Exploration). OTR-A, OTR antagonist.
